# An Enhanced Single Base Extension Technique for the Analysis of Complex Viral Populations

**DOI:** 10.1371/journal.pone.0007453

**Published:** 2009-10-16

**Authors:** Dale R. Webster, Armin G. Hekele, Adam S. Lauring, Kael F. Fischer, Hao Li, Raul Andino, Joseph L. DeRisi

**Affiliations:** 1 Biological and Medical Informatics Program, University of California San Francisco, San Francisco, California, United States of America; 2 Department of Biochemistry and Biophysics, University of California San Francisco, San Francisco, California, United States of America; 3 Department of Microbiology and Immunology, University of California San Francisco, San Francisco, California, United States of America; 4 Department of Medicine, University of California San Francisco, San Francisco, California, United States of America; 5 Howard Hughes Medical Institute, University of California San Francisco, San Francisco, California, United States of America; Institut Pasteur Korea, Republic of Korea

## Abstract

Many techniques for the study of complex populations provide either specific information on a small number of variants or general information on the entire population. Here we describe a powerful new technique for elucidating mutation frequencies at each genomic position in a complex population. This single base extension (SBE) based microarray platform was designed and optimized using *poliovirus* as the target genotype, but can be easily adapted to assay populations derived from any organism. The sensitivity of the method was demonstrated by accurate and consistent readouts from a controlled population of mutant genotypes. We subsequently deployed the technique to investigate the effects of the nucleotide analog ribavirin on a typical *poliovirus* population through two rounds of passage. Our results show that this economical platform can be used to investigate dynamic changes occurring at frequencies below 1% within a complex nucleic acid population. Given that many key aspects of the study and treatment of disease are intimately linked to population-level genomic diversity, our SBE-based technique provides a scalable and cost-effective complement to both traditional and next generation sequencing methodologies.

## Introduction

The identification of specific genetic changes responsible for molecular, cellular, and organismal phenotypes is central to many biological questions. In classical genetic analyses, a phenotype is linked to either the responsible gene or a segregating polymorphism. The power of this approach has recently increased with the development of high-density single nucleotide polymorphism maps in several organisms [1]–[3]. In contrast, many questions require quantification of allele frequency or genetic diversity at the population level. For example, genetic association studies of complex traits often rely on measurements of several independently segregating single nucleotide polymorphisms in diverse populations [4]. In infectious diseases, a single host may harbor a complex mixture of microbes that evolve new traits over short periods of time (e.g. drug resistance or shifts in tropism, see [5]). At the molecular level, genetic rearrangements and somatic hypermutation lead to extensive diversity within B and T cell populations [6]–[8].

A major limitation to such studies is the lack of efficient and economical methods for comprehensive and quantitative characterization of the full range of genotypes in a population. Techniques such as allele specific polymerase chain reaction (PCR), Sanger sequencing, and restriction fragment length polymorphism analysis provide very specific information on a small subset of genotypes. In contrast, single stranded conformational polymorphism (SSCP)[9], heteroduplex mobility (HMA)[10], and MALDI-TOF based assays [11] provide more general information on the entire population. The revolution in ultra high throughput sequencing (UHTS) technology may bridge this gap, but the utility of the current platforms is limited by the relatively high error rate and considerable start up and operating cost [12].

Several groups have successfully used microarrays to measure the frequency of known single nucleotide polymorphisms (SNP) in complex populations. In many platforms, SNPs are identified by differential hybridization of nucleic acid to high-density microarrays tiled with candidate sequences [13], [14]. These techniques often suffer from a high false positive rate and are less accurate than the recently developed arrayed primer extension approach [15]. In array-based primer extension, a sample nucleic acid is hybridized to arrayed oligonucleotides targeting previously characterized SNPs, followed by enzymatic single base extension of the oligonucleotides using the hybridized sample as template. Depending on the identity of the base at a given SNP, a different dye (or hapten) -conjugated chain-terminating dideoxynucleotide is added to the 3′-end the oligonucleotide. In the absence of copy number variation, SNP detection is straightforward, since most alleles are homozygous or heterozygous at any given position with respect to a single individual. While this massively parallel technique allows for simultaneous detection of hundreds to hundreds of thousands of SNPs [16], [17], it requires *a priori* knowledge of the relevant polymorphisms. Many questions in population genetics, however, require a broader, less directed approach to genetic diversity. For example, determining the collective nucleotide sequence of a highly variable population of viruses or T cell receptors can be compared to SNP analysis, with the possibility of a SNP at essentially every position.

Here we describe the development of a platform that utilizes an enhanced version of arrayed primer extension for the measurement of genotype frequencies within a complex population. Because our method relies on single base extension with labeled dideoxynucleotides on replicate arrays, we are able to measure nucleotide frequency without prior knowledge of candidate SNPs. Extensive optimization of this platform allows unbiased detection of single base changes present at 0.3–1% frequency. We demonstrate the performance of our novel mutation distribution analysis of populations (MDAP) microarray by characterizing the mutant spectrum of a *poliovirus* population subjected to a mutagenic drug. Because our system is based on the ubiquitous and economical spotted microarray platform, it is readily adapted to many biological questions and within the reach of a typical university-based laboratory.

## Results

### Overview of the mutation distribution analysis of populations (MDAP) microarray platform

**Figure 1:**
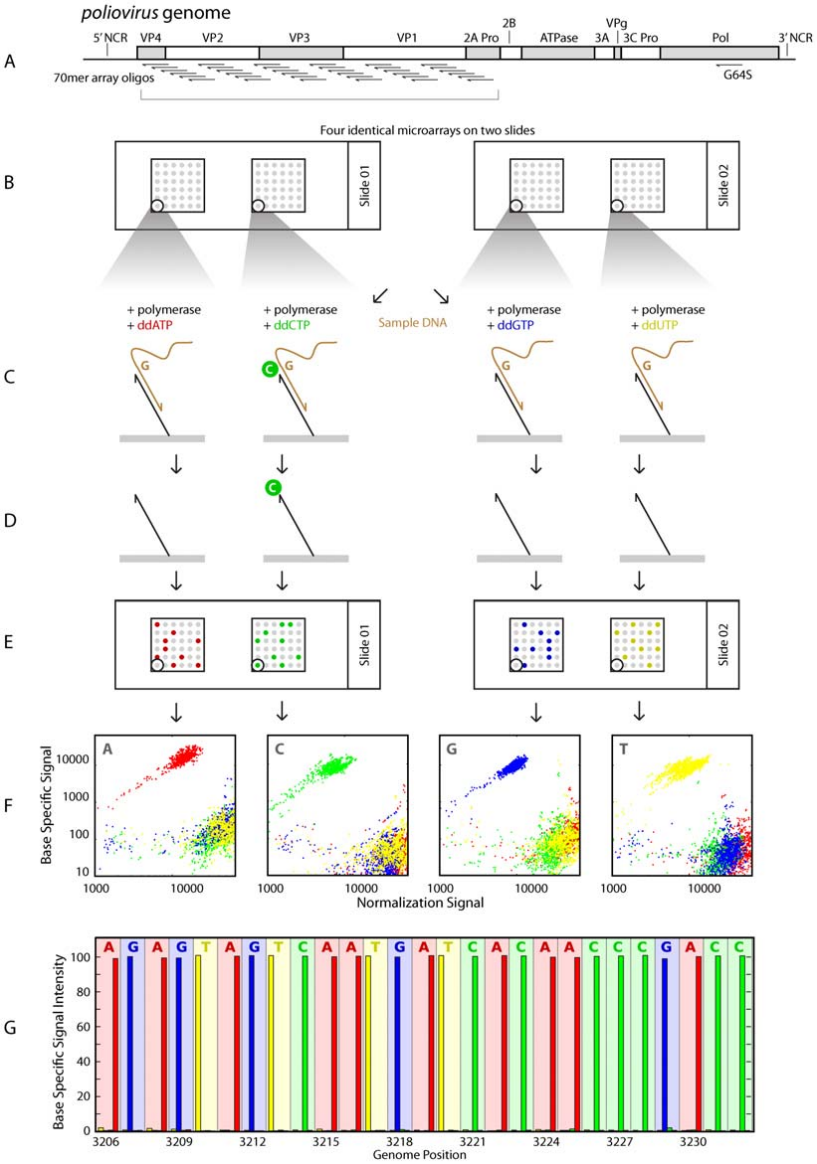
Overview of microarray design and experimental protocol. (A) Schematic of the poliovirus genome. Antisense 70-mer oligonucleotides were designed and synthesized against the capsid region (VP1-4) of the single open reading frame. (B) Four identical microarrays were printed onto two epoxy-coated glass slides, which allowed the oligonucleotides to be covalently coupled to the slide surface. (C) Single stranded DNA was hybridized to all four arrays for a given sample, and enzymatic single base extension (SBE) was performed on each array in the presence of a different fluorescently-conjugated chain terminating dideoxynucleotide. (D) The original sample was removed by high stringency wash, leaving only the oligonucleotides, which either remain unextended or have been extended by a single dye-labeled nucleotide. (E) The arrays were scanned to determine the dye-specific fluorescence intensities for each spot. (F) The base-specific signal from each spot (y-axis) is plotted against a normalization signal (x-axis in F) generated by SBE in the presence of all four ddNTPs. The ratio of base-specific extension over total extension is computed for the analogous feature on all four arrays. (G) Base specific signal as a function of genome position. Background bars represent the expected nucleotide, based on the wild-type *poliovirus* genome. Four foreground bars show the observed frequencies of A(red), C(green), G(blue), and T(yellow) at each position. In this homogeneous population, the “mutant” or incorrect calls reflect the noise of the platform.

We developed a single base extension microarray platform with the goal of determining individual nucleotide frequencies at each genomic position within a complex viral population. For our pilot studies, we focused on the region of the *poliovirus* genome that codes for the structural proteins. We designed overlapping, antisense 70-mer oligonucleotides that hybridize and terminate at each of the 2,643 positions in the capsid portion of the polyprotein open reading frame (Figure 1A). These oligonucleotides were spotted onto epoxy-coated glass slides in triplicate and subsequently covalently coupled to the slide surface (Figure 1B).

We generated single-stranded, positive-strand DNA fragments from poliovirus RNA. Each sample was divided evenly and hybridized to four arrays under identical conditions. We then performed polymerase-based extension on the hybridized fragments using single chain-terminating dideoxynucleotides (ddNTPs, Figure 1C). These ddNTPs were cyanine (Cy)-labeled, and each of the four arrays was exposed to a different mix of one part Cy5 labeled target dideoxynucleotides, one part Cy3 labeled target dideoxynucleotides and two parts each of a mix of the remaining three background ddNTPs. After extension, we removed the poliovirus DNA by boiling in sodium dodecyl sulphate, leaving only covalently bound oligonucleotides, now terminating with labeled ddNTPs (Figure 1D).

We measured nucleotide frequencies at each position of the capsid region by comparing the Cy5 and Cy3 intensities across a set of four arrays. For example, an ‘A’ array received a mixture of one part Cy5 labeled ddATP, one part Cy3 labeled ddATP, and two parts each Cy3 labeled ddCTP, ddGTP, and ddUTP. The “C”, “T”, and “G” three arrays received a corresponding mixture of 3 background nucleotides plus a different ‘target’ nucleotide split evenly between Cy3 (G, green) and Cy5 (R, red) labels. We corrected for differential incorporation rates and fluorescence intensities using a set of oligonucleotides expected to extend Cy3 and Cy5 labeled ddNTPs with equal frequency. The global dye-specific intensities were normalized such that the median values for each were equal on these control oligonucleotides. After normalization R*_n_* accounts for 50% of the total florescence derived from the incorporation of nucleotide *n*, and G*_n_* + R*_n_* represents the total florescence observed from extension of all four nucleotides. It follows that equation (1) below closely approximates the fraction of oligonucleotides F within a given spot that were extended by the target nucleotide *n*.
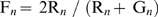
(1)


Since equation (1) represents the fraction of oligonucleotides within a spot that were extended with the labeled nucleotide of interest on that array, the sum of this ratio across all four arrays was normalized to one to eliminate any global dye bias.
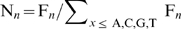
(2)


Three identical spots assaying each position *p* within the genome were used to calculate the median value of N*_n_*. This value was used as the final frequency of nucleotide *n* in the population at position *p*, N*_n,p_*.
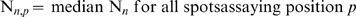
(3)


### Optimization of MDAP array to a detection threshold below 1%

During the process of design, implementation, and optimization of the MDAP array, we identified many sources of experimental noise that were either minimized or eliminated altogether. The signal to noise ratio of the array served as a measure of array quality and was the major target for optimization. Because any natural *poliovirus* population is heterogeneous, we used *in vitro* transcribed RNA derived from a plasmid *poliovirus* clone as a reference set [18], [19]. While T7 RNA polymerase does have an error rate of approximately 10^−5^, any mutants generated during *in vitro* transcription would be present at a frequency of ∼0.001% and below the limit of detection for our assay [20], [21]. In our analyses, any signal corresponding to incorrect bases was therefore considered to be the background noise of the assay. In order to achieve the final design goal of sensitivity below 1%, four major components of the experiment were optimized: surface chemistry, oligonucleotide properties, hybridization, and extension.

Although we found that slide chemistry did not directly affect the signal to noise ratio, few slide chemistries could survive the required extension and stringent wash steps of the assay. For example, poly-l-lysine coated slides deteriorated during the polymerase-based extension step. Others were unable to withstand the final wash in boiling sodium dodecyl sulphate, which removed all nucleic acid not covalently linked to the slide surface. Of the slide chemistries tested (poly-l-lysine, epoxy, aldehyde, and amino-silane), epoxy slides exhibited the best combination of spot morphology and stability. We also tested several print buffers, including 3xSSC, Formamide, Betaine, nextSpot, phosphate, Tween20 and sarcosyl, with 3xSSC providing the best combination of performance and utility.

We empirically determined the optimal oligonucleotide length. Oligonucleotides assaying the same genomic position were synthesized at lengths of 20 to 70 nucleotides in ten nucleotide increments. We designed three sets to interrogate different positions, and printed them in quadruplicate on the array. Hybridization and extension were performed with a homogeneous *poliovirus* population and the raw intensity of the extension signal recorded for all 72 observations (3 positions x 6 oligonucleotides of varying length x 4 spots for each oligonucleotide). We detected little variation in median signal intensity among 50-, 60-, and 70-mers but observed a greater than 50% reduction in signal with oligonucleotides shorter than 50 mers (Figure S1). These data are in accordance with previously published reports on the relationship between hybridization performance and oligonucleotide length [22], [23]. We therefore used 70-mer oligonucleotides in all subsequent experiments.

We found it necessary to minimize two other sources of oligonucleotide noise. For two to three percent of the oligonucleotides, self-hybridization or dimerization provided a valid template for extension. This resulted in very strong base-specific noise. To eliminate this technical artifact, we identified oligonucleotides prone to self-hybridization using an Mfold-based algorithm. Mutations were then introduced into array oligonucleotides that would preserve appropriate hybridization but prevent self-extension ([24],Figure S2). This technique proved successful, reducing the noise on these oligonucleotides to background levels. We also identified an unexpected source of noise related to the oligonucleotide manufacturing process. Based on observed oligonucleotide-specific patterns of noise, we inferred that greater than one percent of oligonucleotides within each spot were missing one or more bases at their 3′ termini. This resulted in aberrant extension in place of the absent nucleotide at a frequency of one to three percent (Figure S3). Since these oligonucleotides were synthesized from 3′ to 5′, the missing nucleotides were a result of failures in the initial stages of synthesis. We therefore tested oligonucleotides synthesized by an alternate process (base-specific rather than universal support, see Materials and Methods) and observed a 29% reduction in mean noise levels across the array (Figure S4).

We next optimized the hybridization and extension steps. Both single-stranded and double-stranded nucleic acid have been used successfully in microarray experiments. We found that single-stranded nucleic acid exhibited both increased raw extension intensities and decreased noise ratios compared to double-stranded template (Figure S5). The difference is striking, with an approximately 30-fold increase in signal and no significant change in the corresponding noise levels. Hybridization time also had a significant impact on global oligonucleotide noise. By reducing the hybridization time from 12 hours to 30 minutes, we decreased the absolute median noise level from 1.8% to 0.4% of the signal, a relative reduction of 78% (Figure S6). Although decreasing the duration of the extension reaction from one hour to five minutes did not produce a similar improvement (Figure S7), these two protocol changes did decrease the per sample assay time by 80%.

By optimizing the parameters above, we were able to correctly determine the correct base at each position across the viral capsid with a median signal to noise ratio of 250∶1 for a homogeneous population (Figure S8). In a typical experiment, 70% of oligonucleotides had a signal to noise ratio greater than 100∶1, corresponding to a sensitivity of 99%. Many other experimental parameters were tested throughout the optimization process (Table S1), with little or no effect on array performance. One important source of noise is experiment-specific and dependent on the relative frequencies of mutations in the population. If a single mutation rises to high levels within a population, it can affect the performance of oligonucleotides assaying nearby positions. Template molecules containing the mutation will hybridize correctly, but mismatches between the template and the 3′ end of an oligonucleotide decrease the efficiency of the single base extension (Figure S9). Control experiments in which 100% of the population contained a specific mutation showed that extension was nearly abolished when mismatches occurred at the neighboring position. We observed smaller effects for mismatches up to six nucleotides (approximately one half-turn of the primer-template duplex) from the 3′ end of the oligonucleotide (data not shown). This effect can be abrogated by designing contingency oligonucleotides for a given position that include nearby polymorphisms known to be present in the sampled population.

### MDAP can accurately measure mutation frequency in a controlled population

**Figure 2:**
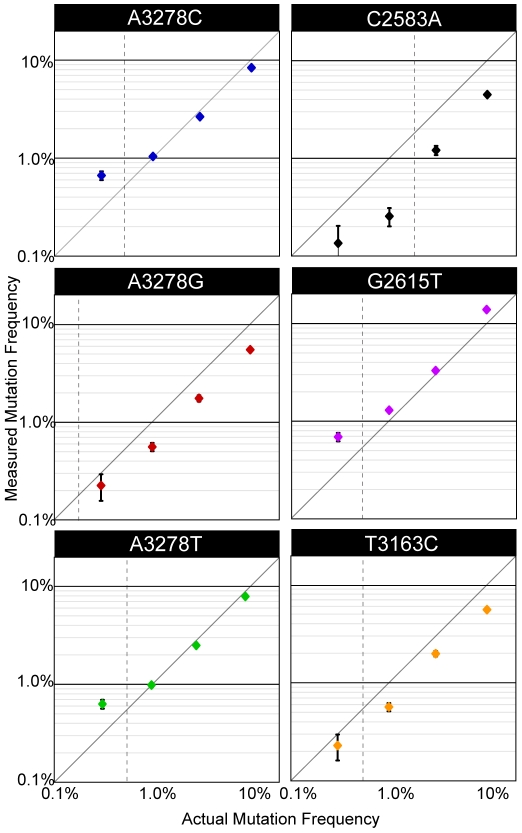
Array accuracy and sensitivity. The mutation frequencies measured by MDAP (y-axis) are plotted against the actual (expected) mutation frequency (x-axis). Genomes were mutated at single positions and mixed together at frequencies of 0.3%, 1%, 3%, and 10%, with the remainder of the population in each case derived from an unmutated sample. Two array replicates were performed to generate error bars representing the standard deviation of six total observations (three oligonucleotide replicates on each of two array replicates). Titles indicate wild type base, genome position, and mutated base. The vertical dotted line in each panel indicates the significance cut-off based on z-scores derived from a homogeneous control sample. Data points to the left of the dotted line are not statistically significant.

We tested the sensitivity and accuracy of the MDAP platform by measuring the frequency of specific mutants within a defined population. We introduced a set of point mutations into the capsid region of the *poliovirus* genome and generated the corresponding *in vitro* transcribed RNA. These mutated genomes were combined with each other and unmodified poliovirus RNA such that each mutant RNA would be present at a frequency of 10%. We then diluted this pool serially with unmodified RNA, to obtain mutant frequencies of 3.16%, 1.0%, and 0.316%. Single-stranded DNA was prepared from each of the four dilutions. As controls, we used DNA prepared from two samples of unmodified *poliovirus* RNA. We then processed these samples using the MDAP array. Mutation frequencies measured from the array are shown in Figure 2. The accuracy varied across the sample set, with a mean observed frequency in the 10% mixture of 8.06% and a standard deviation of 3.6%. Predicted frequencies of the mutations in diluted samples were very consistent, with R squared values of linear fits to each dilution series ranging from 0.991 to 0.999.

The sensitivity of the array was measured by comparing each of the mutant-templated arrays to those templated by unmodified, wild type nucleic acid. The distribution of mutation frequencies observed for a given spot in the control arrays was assumed to be noise for all non-wild type nucleotides. This background signal was used as a baseline from which to calculate a standard z-score for each observation on the experimental arrays. The minimum z-score across three spot replicates on each of two array replicates for each sample was taken as the significance score for a given nucleotide and position. Introduced mutations were considered to be detectable if their z-score was higher than all of the significance scores for unexpected mutations (false positives) on the control samples. Data points to the left of the dotted line in Figure 2 denote introduced mutations whose significance score fell below those of the most significant false positives in the sample. At a frequency of 0.3%, only one of the introduced mutations (A3278G) was detectable. When the introduced mutations were mixed at 1%, five of the six had z-scores greater than any unexpected mutation. These data suggest that the average limit of detection for the MDAP array is between 0.3% and 1%. The observed variability in position-specific sensitivity is an inherent feature of hybridization-based methodologies and reflects the nonrandom influence of natural sequence variation.

### Monitoring an evolving viral population with MDAP

To determine the ability of the array to monitor genome-wide changes in a complex population over time we investigated the effects of the nucleoside analog ribavirin on a *poliovirus* population. We and others have previously shown that ribavirin is an RNA virus mutagen [25]. We generated a wild type *poliovirus* population by transfecting *in vitro* transcribed RNA into HeLa S3 cells. Viral supernatants were harvested from transfected cells and passaged once at high multiplicity of infection (MOI) to generate a population that served as the passage 0 (P0) viral stock for the experiment. We used this stock to infect four cell preparations exposed to varying concentrations of ribavirin (0 µM, 100 µM, 400 µM, and 1000 µM). Passage 1 (P1) virus was harvested from each sample upon cytopathic effect and used to infect a fresh monolayer of cells at the same drug concentration. The virus resulting from this second round of infection was termed P2. We analyzed this set of samples to identify the mutations present in the population as a function of drug concentration and time.

**Figure 3:**
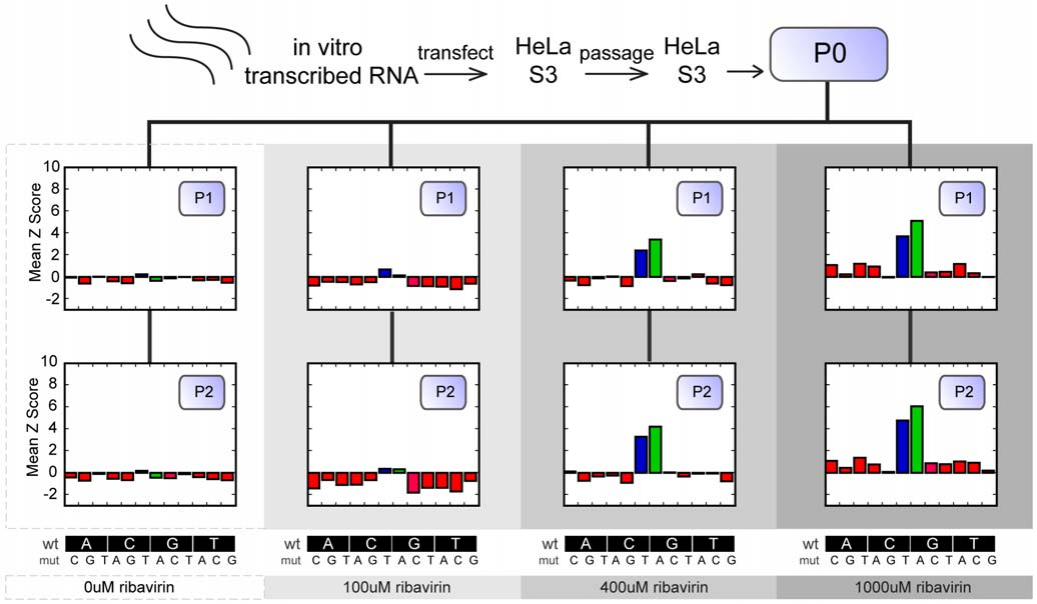
Global monitoring of mutations in a viral population. A wild type population was generated by transfection of *in vitro* transcribed genomic RNA into HeLa S3 cells. This supernatant was passed once on a fresh monolayer to derive a P0 stock. This population was serially passaged at MOI 0.1 in the presence of 0 µM, 100 µM, 400 µM or 1000 µM of the antiviral nucleoside analog ribavirin. The significance (standard z-score) of base changes as compared to the reference (eight replicate observations of the P1 0 µM ribavirin sample) was recorded and the mean significance scores were plotted for every observed mutation type. A mean z score greater than zero indicates increasing average significance of the given mutation type. Values less than zero indicate that the observed frequencies are below the assay background. Specific classes of mutations are shown at the bottom of the figure. The two types of mutations showing the most significant change throughout the experiment are colored blue (C-to-T) and green (G-to-A).

Because all *poliovirus* populations are heterogeneous [18], [19], we used virus passaged in the absence of drug as a reference. This control allowed us to distinguish mutations induced by ribavirin from standing genetic variation. Based on data derived from two array replicates of this sample, we calculated a significance score (standard z-score) to estimate the relative likelihood that a given observation from other passages or drug concentrations fell outside the standard variation of the base-specific signal for a viral population. Using this method, small changes in array signal were assigned z-scores to represent our confidence that the observed change in signal reflected actual changes in mutation frequency as opposed to oligonucleotide noise or “background” viral genetic diversity. A high z-score would suggest that a given mutation was overrepresented in the population and correlated with drug treatment or passage. To quantify global changes in mutation frequency, we plotted the mean z-score for each of the twelve possible nucleotide changes as a function of time and drug concentration (e.g. A to C, A to G, A to T, C to A, C to G, C to T, etc.). A clear overrepresentation of cytosine and thymine (C to T) and guanine to adenine (G to A) mutations was apparent in viral populations passaged in ribavirin, and their frequency increased with drug concentration (Figure 3). We observed little change in the frequency of other mutation classes across the entire capsid sequence. These data are consistent with ribavirin's known mode of action and demonstrate the ability of the MDAP technique to identify and characterize the spectrum of mutations in a complex population [25], [26].

Quasispecies theory predicts that RNA viral populations are robust to the effect of mutation, and that many of the mutations in viable viruses do not code for amino acid changes [27]. According to this model, the virus would evolve over a neutral fitness landscape and many of the mutations would be synonymous in nature. We therefore estimated the ratio of nonsynonymous to synonymous changes within our mutagenized population as measured by MDAP. By assuming that the vast majority of observed mutations were single nucleotide changes within a codon, each mutation was assigned to the NS (nonsynonymous) or S (synonymous) class of mutations, and the average z-score of each class was computed for every codon. In P1, the average z-score of S mutations was not significantly different than that of NS mutations (p<0.068, 0.13, 0.28 for 100, 400, 1000 µM ribavirin, respectively), suggesting that there was no bias for noncoding changes at early passages. In P2, however, the average z-score of S mutations was significantly higher than the average score of NS mutations (p<0.08, 0.006, 0.07). These data indicate that synonymous changes accumulate over time, consistent with negative selection against coding changes. To determine whether there were ‘hot spots’ of selective pressure, we calculated a per codon estimate of the selective pressure as the difference between the average z-score of nonsynonymous mutations and the average z-score of synonymous mutations within the codon. Ann excess of nonsynonymous mutations would give a positive value and suggest positive selection at that position. Using this metric, we found no evidence for nonsynonymous “hotspots” across the capsid region of the genome (Figure S10). The observed variability in the z-scores for different SNPs likely reflects the fact that certain sites are subject to reduced selective pressure under our experimental conditions. Mapping of these highly significant SNPs onto the surface of the capsid did not, however, suggest any higher order, biological correlations. Together these data indicate that the majority of the viable mutants are selectively neutral and that ribavirin acts in a random fashion across the genome.

**Figure 4:**
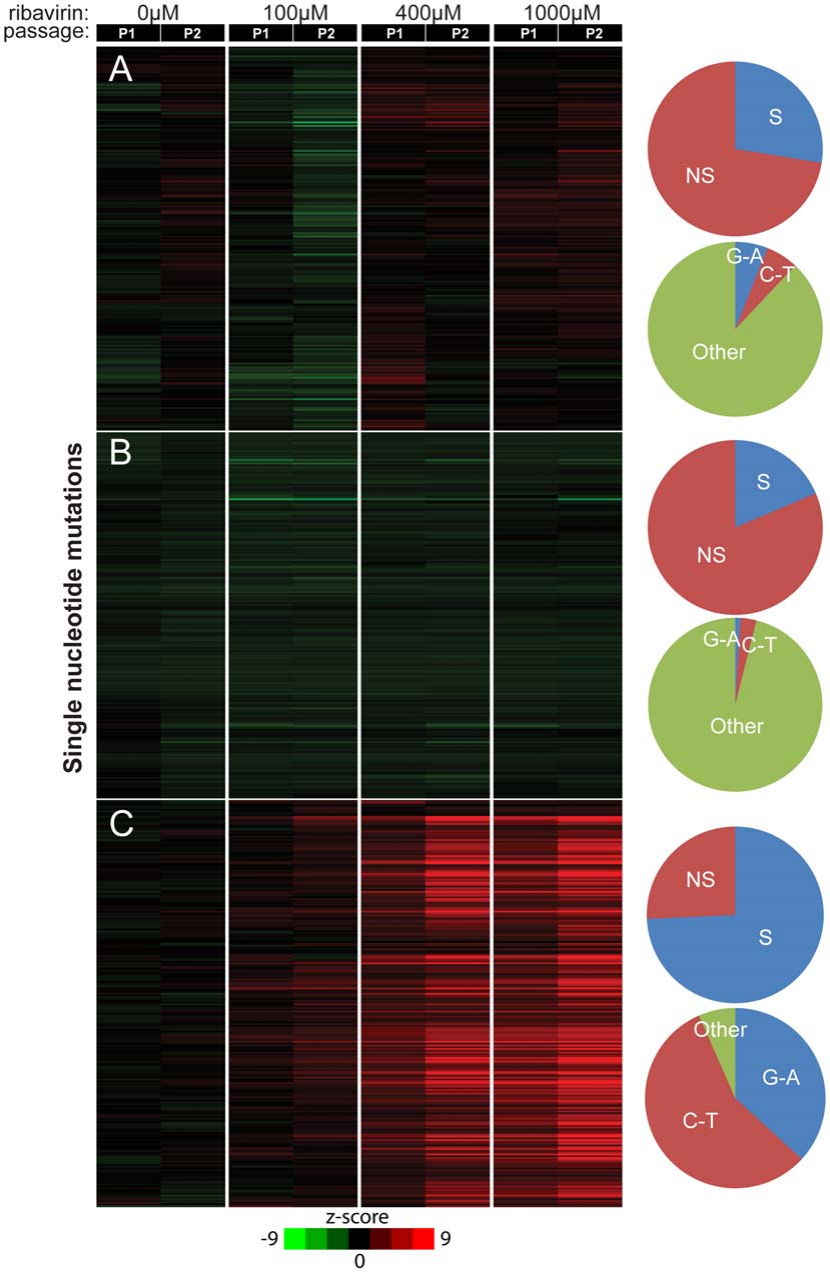
Mutations with Similar Behavior Reveal Selective Pressure. Z-scores representing the significance of mutational changes away from P1 in the absence of drug were clustered, and clusters showing interesting behavior were further analyzed for selective pressure and mutation type. Each row in the cluster represents a single mutation, and each column represents a single passage. Green bars denote mutations that are decreased in frequency compared to P1 no drug, black indicates no significant change, and red bars indicate mutations that have increased in frequency. Pie charts show the composition of nucleotide changes and synonymous or nonsynonymous nucleotide changes in the indicated clusters A–C.

To better characterize the nature of selection in drug-treated viral populations, we performed cluster analysis of the changes observed in the ribavirin samples. Mutations were clustered according to their relative z-scores in passages one and two (Figure 4). We identified a number of strong, monotonically increasing mutations that formed a particularly distinct cluster (Figure 4C). Consistent with our global analysis of mutation frequency, nearly all of these mutations were G-to-A or C-to-T changes. To determine whether these mutations were increasing from passage to passage due to low levels of fixation in the population, we computed the frequency of synonymous and nonsynonymous changes within the cluster. While 20% of all possible mutations in the region analyzed by this array are synonymous changes, 74.3% of the 214 mutations in this cluster were synonymous. (p<0.01). Therefore, the vast majority of mutations that accumulate in the population over time do not code for amino acid changes and are less likely to alter viral fitness. Our cluster analysis also indicates that there is strong negative selection against coding changes, which is consistent with our prior work on lethal mutagenesis of RNA viruses [28].

### Observing the Dynamics of Ribavirin Resistance

**Figure 5:**
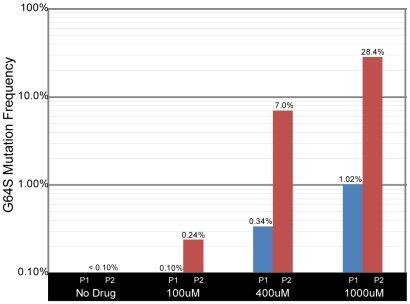
Measuring the frequency of the ribavirin resistance mutation. The frequency of a G-to-A mutation at position 6176 of the *poliovirus* genome, which is known to confer resistance to ribavirin, is shown on a log scale. Passages 1 and 2 (Blue and Red) are shown for each concentration of ribavirin (0, 100, 400, 1000 µM).

While drug resistant variants are known to exist in viral populations prior to drug exposure, conventional sequencing-based assays are only able to detect mutants present at a high frequency. The sensitivity and low detection threshold of the MDAP platform offer an opportunity to observe the dynamics of drug resistance at a relatively early stage. We and others have identified a point mutation within the *poliovirus* 3D polymerase gene that mediates ribavirin resistance [29], [30]. This mutation in the RNA-dependent RNA polymerase (glycine to serine at amino acid 64) results in increased polymerase fidelity, and is thought to decrease the rate at which the polymerase incorporates ribavirin when replicating the *poliovirus* genome. To quantify the levels of this drug resistant mutant in the populations described above, an additional oligonucleotide was designed to assay position 6176 of the *poliovirus* genome (Figure 1A). The measured prevalence of this mutation is shown in Figure 5. In the presence of 100 µM ribavirin, the frequency of the G64S mutation increases slowly, reaching 0.24% of the population after two passages. In contrast, by passage two in 400 µM ribavirin, the G64S mutation is present in 7% of viral genomes. Not surprisingly, the G64S mutation increases in frequency even more quickly in the presence of 1000 µM ribavirin, reaching 28% of the population after just two passages. These results highlight the complex nature of drug resistance and the ability of the MDAP platform to identify minority variants within a viral population.

### MDAP analysis

In addition to the MDAP array described here, we have also developed freely available companion software to facilitate rapid and detailed analysis of array results. The open source MDAP software package provides an intuitive graphical user interface, allowing users to load GPR (GenePix Results) files from multiple arrays and generate sample files containing all data relevant to a single sample. Data can be explored through a series of automatically generated graphs, allowing the user to quickly examine data by genome position, fluorescence intensity, and mutation frequency at the level of a single array, sample, or even a reference set of samples. Mutation frequencies, as well as z-scores when a reference set is available, can be exported to text files for further analysis in other programs. The MDAP software package is implemented in python, and can be used on all major computing platforms. The source code is available at http://derisilab.ucsf.edu/software/MDAP/.

## Discussion

Many techniques for the study of complex populations provide either specific information on a small number of variants or general information on the entire population. To address this shortcoming, we have developed an economical and scalable method that provides comprehensive and unbiased information on allele frequency across an entire genome. Our novel microarray platform builds upon previously described single base extension methods employed for SNP detection [15], [16]. By using labeled dideoxynucleotides in our primer extension and replicate arrays, we were able to identify mutations over a 2643 nucleotide region without *a priori* knowledge of specific polymorphisms. Our analyses of a *poliovirus* population evolving under the pressure of a mutagenic drug demonstrate the sensitivity of the platform for minority genotypes in the background of a complex population. In this biologically relevant application, we found that the array correctly identified mutations characteristic of ribavirin's mode of action and detected drug resistant variants at an early stage.

### A system for accurate measurement of low frequency polymorphisms

We devoted considerable effort to optimizing each step of the MDAP protocol. As is the case for many microarray platforms, we found that proper oligonucleotide design is critical. While 70-mers provided the best signal to noise ratio, our data suggest that shorter oligonucleotides could provide adequate performance at a lower cost. Investigators interested in adapting our platform to other systems should also use appropriate algorithms to identify candidate oligonucleotides that can dimerize or adopt secondary structures. Surprisingly, we found that 3′ to 5′ oligonucleotide synthesis resulted in aberrant extension of incomplete oligonucleotides and a significant amount of noise. We therefore caution others to work with their suppliers to identify the appropriate oligonucleotide synthesis method for this platform. Optimization of hybridization and extension times yielded smaller but significant improvements in MDAP performance. Together, these changes resulted in a signal to noise ratio of 100–250, which translates to a per SNP detection threshold of between 0.4% and 1%. Our ability to identify specific point mutants within a complex population validated the assay's ability to reproducibly detect polymorphisms present at less than 1%.

At the lower limit of detection, we found that the sequence context of a given SNP could have a significant effect on our sensitivity and specificity. For example, our platform was less sensitive for minority variants in which two mutations are separated by less than six bases, and we were unable to detect variants with neighboring mutations (Figure S9 and data not shown). While we cannot say how often this would occur in a given experimental setting, pilot experiments with clinical isolates of poliovirus indicated that certain naturally occurring variants would escape detection. The impact of context could potentially be mitigated by the inclusion of additional oligonucleotides that would specifically interrogate known minority variants. This process would, of course, require *a priori* knowledge of the relevant mutations, and would be difficult to apply to clinical samples.

### Characterizing the mutant spectrum of an evolving viral population

We examined the ability of the MDAP platform to measure changes in the capsid gene of *poliovirus*, a model RNA virus. The *poliovirus* RNA-dependent RNA polymerase exhibits low fidelity with measured mutation rates of 10^−4^ to 10^−5^ mutations per nucleotide per replication cycle. This high mutation rate is characteristic of RNA viruses and leads to extraordinary genetic diversity in RNA virus populations. These populations are termed “quasispecies,” and a growing body of evidence suggests that the spectrum of mutants within the population determines its phenotype [19]. Accordingly, population-level responses to selective pressures, such as drug treatment, are complex. This system, therefore, offered a unique test of the MDAP platform. In ribavirin treated populations, we observed an overrepresentation of G-to-A and C-to-T mutations, consistent with the drug's known mode of action [25]. In early passages, many of these polymorphisms were present at low frequency and would escape detection by conventional methods. Cluster-based analysis of the array data allowed us to infer evolutionary dynamics from base-specific mutation frequencies within the population. Most of the mutations were synonymous, suggesting that the vast majority of coding changes result in inviable genomes. Our poliovirus experiments also allowed us to characterize the emergence of antiviral drug resistance. We were able detect drug resistant variants at early passages, well before they dominate the population.

We developed the MDAP platform as a cost-effective method for studying the genetic diversity of viral populations. This diversity is characteristic of RNA viruses, and the degree to which minority variants influence the adaptability and phenotype of the population has remained a much debated, but largely unanswered, question. While we have achieved our primary goal of identifying SNPs present at a 0.3–1% frequency, the present MDAP platform has several limitations. If rare variants in a population are present at levels below 0.3%, they will likely escape detection, yet this may include variants that could affect population dynamics. This problem is not unique to our system, however, as current deep sequencing technologies have a similar detection threshold, depending on platform and depth. Our SNP detection array provides little information on linkage among mutations or overall haplotype, which is also a problem common to many resequencing platforms that rely on relatively short reads. Finally, we have found that highly divergent variants will escape detection because of inefficient extension from the consensus oligonucleotides on the array. While this was not a problem in our experiments, it may be difficult to apply the MDAP platform to analyze highly variable genomic regions of complex populations. Given these limitations, the “sweet spot” for MDAP analysis is for populations of sequences derived from a single consensus and that harbor variants at a frequency of at least 0.3%.

### Comparison to other microarray-based resequencing platforms

There has been an explosion in high-throughput genotyping technologies over the last decade. The earliest platforms were built on standard microarray technology and relied on high-density arrays with four degenerate probes for each interrogated position in a given gene [13], [14], [31], [32]. Differential hybridization among these probes was then used to determine the base at each position. While advances in photolithography and solid phase oligonucleotide synthesis technologies improved the amount of sequence that could be analyzed with such arrays, this strategy was plagued by relatively high background and low specificity, as it was highly sensitive to sequence context. More recent strategies, which rely on single base extension, require fewer array oligonucleotides per position, increasing the number of SNPs that can be detected on a single microarray [15], [16]. Although the SBE approach offers improved scale, performance was optimized mainly to discriminate between homozygosity and heterozygosity for the purpose of high throughput genotyping. While the MDAP platform is similar in design to SBE assays, its improved sensitivity and specificity allow for accurate quantitation of low frequency polymorphisms within a mixed population. Like other SBE platforms, it is hampered by many of the same limitations - difficulties with long repeats, reduced sensitivity for neighboring mutations, and reliance on a known reference sequence relatively similar to the genome to be assayed. Because MDAP, as designed, relies on spotted microarrays, its resequencing capacity is also lower than that of commercial SBE platforms, which use higher density, synthesized arrays or beads to achieve greater throughput. The choice of platform, therefore, depends on the type of information required for a given study.

### MDAP is extensible to a broad range of biological applications

Although the MDAP array described here was designed to assay only the viral capsid sequence of *poliovirus*, it can be easily extended to monitor the entire genome. Standard spotted microarrays may contain up to 50,000 features; thus, this platform provides enough features to assay the entire genome of many RNA viruses with three-fold or more redundancy. Moreover, this platform is easily adapted to assay a wide range of populations. The number of microarray-based assays has risen rapidly in the past few years, resulting in decreased cost and increased access for researchers to microarray-related resources. As a result, most research institutions are equipped with the basic microarray functionality required for the assay described here. We have also improved access to this technology by developing a data analysis software package that is freely available to the scientific community.

The cost of oligonucleotide synthesis is the main barrier to widespread use of the MDAP platform. This initial cost varies linearly with the length of the target sequence, and is currently estimated at $52,087 USD for a complete set of oligonucleotides designed against a typical picornavirus genome (7,441 nucleotides, Table S2). For comparison, current startup costs for the ultra high throughput sequencing (UHTS) approach to complex population resequencing would be approximately $500,000, not including yearly maintenance fees (Genome Analyzer, Illumina, Inc). After the initial startup costs, the recurring cost of the MDAP array is comparable to that of a typical UHTS platform (Table S2). For smaller genome sizes (around 14 kb), the two assays are similarly cost-effective. For larger genomes UHTS based analysis becomes two or three times as expensive as the microarray, up to the microarray limit of 50,000 features. With similar per sample costs and significantly lower initial cost, the microarray platform proposed here will likely be preferable to UHTS for many resequencing applications. Both techniques generate mutation frequencies for each position assayed, and both share limitations with respect to detection of co-occurring mutations. One advantage of this platform over UHTS is a significantly lower cost of failure. A single run of the Genome Analyzer costs around $4,400, and common failure modes affect either a single lane ($550), or the entire run. Most failure modes of the array described here impact only a single sample, for minimal financial impact ($100).

In conclusion, we have designed, implemented and demonstrated a single base extension microarray platform optimized specifically for use in genotyping complex nucleic acid populations. Presently, it is one of the more cost effective methods for such studies, although the ongoing revolution in UHTS may change this equation in the future [12]. The MDAP array can be applied to any organism and condition from which a representative sample of single stranded DNA can be extracted. Because it can assay tens of thousands of genomic positions simultaneously, our platform is well suited to answer biological questions requiring accurate and sensitive detection of single nucleotide mutational frequencies at a population level.

## Materials and Methods

### Oligonucleotide design

Oligonucleotides were designed to hybridize to the sense strand of the capsid region of the *poliovirus* type 1 (Mahoney) genome. One oligonucleotide was designed against each 70 bp region of the capsid, with adjacent oligonucleotides overlapping by 69 nucleotides. A single additional oligonucleotide was designed to hybridize to the 70 nucleotides 5′ of the known ribavirin resistance mutation (position 6176) [19], [30]. Oligonucleotide secondary structure and dimerization was predicted using MFold v3.1[24], and predicted base-pairing of 4 or more nucleotides at the 3′ terminus of the oligonucleotide was disrupted by mutating the position predicted to pair with the 3′ most base of the oligonucleotide to its complement.

### Microarray fabrication

The 70-bp oligonucleotides were synthesized (Invitrogen, Carlsbad, CA), resuspended in 3 x SSC to a final concentration of 20 µM and spotted onto epoxysilane-coated microscopic slides (Schott, Louisville, KY). All oligonucleotide sequences are available at http://derisilab.ucsf.edu/data/MDAP/.

### Cells and viruses

HeLa S3 cells (ATCC, CCL-2.2) were propagated in DMEM/F-12 media (Invitrogen, Carlsbad, CA) supplemented with 10% fetal bovine serum (SIGMA, St. Louis, MO). Wild-type poliovirus type 1 Mahoney was generated by electroporation of HeLa S3 cells with *in vitro* transcribed viral genomes that were derived from cloned infectious cDNA using T7 RNA polymerase. For virus passage approximately 10^6^ cells were plated in each well of a six-well plate 12 h prior to infection. One hour prior to infection, cells were pretreated with ribavirin (SIGMA, St. Louis, MO) or left untreated. Mutagenesis experiments were done at a multiplicity of infection (MOI) of 0.1 at 37°C. Cells and tissue culture supernatants were harvested at complete cytopathic effect (CPE) and subjected to three cycles of freezing and thawing. Virus suspensions were clarified by centrifugation and stored at −80°C until further use.

### Template generation

Total RNA was isolated directly from virus suspensions by TRIzol (Invitrogen, Carlsbad, CA) extraction. Briefly, 500 µl virus suspension were thoroughly mixed with 500 µl TRIzol and, after addition of 100 µl chloroform, spun at 12,000 g for 15 min at 4°C. The aqueous phase was extracted with chloroform and the RNA was precipitated with 2-propanol in the presence of 20 µg glycogen (Fermentas, Glen Burnie, MD). Pellets were air dried and resuspended in nuclease-free water.

For first strand cDNA synthesis, 20 ng of total RNA were mixed with 2 pmol each of primers V_3_-Oligonucleotide(dT)_21_ as well as M.3666REV (5′-TGGTACCTAGCTG-3′), M.3699REV (5′-GATGCGAATCCATG-3′), M.3726REV (5′-CTGAGTATGCCACC-3′), M.3764REV (5′-CACCAGCAGTAATG-3′), M.3783REV (5′-AATGCAACCAACC-3′) and M3840REV (5′-GTGATGCCTTGTTC-3′), which bind to the *poliovirus* RNA immediately downstream of P1. The mixture was heated to 65°C for one minute and then chilled on ice for one minute. Reverse transcription was started by adding MonsterScript 2x cDNA premix (containing buffer, dNTPs and betaine) and 12.5 U RNaseH-deficient MonsterScript reverse transcriptase with RNase inhibitor (EPICENTER, Madison, WI). The total reaction volume was 5 µl. Reverse transcription was carried out for 5 min at 37°C, 5 min at 42°C and 1 h at 60°C followed by heat inactivation of the enzyme.

Sequences corresponding to the poliovirus capsid and RNA-dependent RNA polymerase were amplified between primer pairs M.501FOR and M.3634REV\T7\ (5′-GATTGGCCTGTCGTAA CGC-3′; 5′-TAATACGACTCACTATAGGGCATGTACTGGAACGTTGGG-3′) and M.5781FOR and M.6811REV\T7\ (5′-GTGCTGTGACTGAACAGGGG-3′; 5′-TAATACGAC TCACTATAGGGGTACAGGTGGTGTCAGTGG-3′), respectively. Amplification reactions consisted of up to 2.5% first-strand cDNA, 1 x Herculase buffer, 200 µM of each dNTP, 200 nM of each primer and 0.05 U/µl Herculase Hotstart DNA polymerase (Stratagene, La Jolla, CA). Thermal cycling was carried out for 30 cycles of 30 seconds at 95°C, 30 seconds at 60°C and 1 minute per kb of amplification product at 72°C.

PCR products carrying the T7 RNA polymerase promoter were used without further purification to generate complementary RNA (cRNA). *In vitro* transcription reactions consisted of up to 10% PCR product, 1 x *in vitro* transcription buffer (80 mM HEPES-KOH pH 7.5, 24 mM MgCl_2_, 2 mM spermidine, 40 mM dithiothreitol), 4 mM of each NTP, 0.4 U/µl RNaseOUT recombinant ribonuclease inhibitor (Invitrogen, Carlsbad, CA) and T7 RNA polymerase (prepared in house). After incubation at 37°C for 4 hours, template DNA was degraded by adding 0.2 U/µlRNase-free DNase I (Roche, Indianapolis, IN) with continued incubation at 37°C for 20 minutes. The cRNA was diluted 1∶6 with nuclease-free water and ammonium acetate to a final concentration of 0.5 M ammonium acetate and extracted with phenol:chloroform:isoamyl alcohol (25∶24∶1; Invitrogen, Carlsbad, CA), followed by a chloroform extraction. The cRNA containing aqueous phase was precipitated in the presence of an equal volume of 2-propanol. Pellets were air dried and resuspended in nuclease-free water.

Single-stranded microarray template DNA was generated by reverse transcription of the cRNA in the presence of random hexamers. To this end, 6 µg cRNA were mixed with 300 µg random hexamers in a total volume of 30 µl, heated to 70°C for five minutes and chilled on ice for one minute. Reverse transcription was started by adding 30 µl reverse transcription premix, containing 2x reverse transcriptase buffer (100 mM Tris-HCl pH 8.3, 150 mM KCl, 12 mM MgCl_2_), 1 mM of each dNTP, 10 µM dithiothreitol, 1.33 U/µl RNaseOUT recombinant ribonuclease inhibitor (Invitrogen, Carlsbad, CA) and reverse transcriptase (prepared in house). The mixture was incubated for 10 minutes at 20°C and subsequently for three hours at 42°C. After heat inactivation of the enzyme, the input cRNA was hydrolyzed for 20 minutes at 65°C in the presence of 0.2 mM NaOH and 20 mM EDTA. After adding 1 Vol. 1 M HEPES-KOH pH 7.5 and 0.2 Vol. 3 M sodium acetate pH 5.2, single-stranded microarray template DNA was purified over a QIAquick column (QIAGEN, Valencia, CA) following the manufacturer's instructions.

### Hybridization and single base extension

Two 0.2 mm strips of microtiter plate sealing film were applied along the long edge of the glass slides leaving narrow spaces between the strips and the microarrays. Prior to hybridization of single-stranded template DNA to the microarrays, unreacted epoxy groups were blocked by incubating the slides for 20 minutes at 50°C in 0.1 M Tris-HCl pH 9.0 containing 50 mM ethanolamine and 0.1% SDS. The volume of blocking solution should be at least 20 ml per slide. After blocking, slides were rinsed in six changes of deionized water and dried by centrifugation.

Four identical microarrays (two per slide) for every viral population to be sequenced were placed into a hybridization chamber (Die-Tech, San Jose, CA). Using the two film strips as support, glass cover slips were positioned above each microarray. For each array, 30 µl hybridization solution containing 1 µg single stranded template DNA in 3 x SSC, 25 mM HEPES-KOH pH 7.5 and 0.25% SDS were heated to 95°C for three minutes and then allowed to cool to ambient temperature. The hybridization solution was injected between the microarray slide and the cover slip and hybridization reactions were incubated at 65°C for 10 minutes. Slides were washed for 10 minutes at 60°C in 0.5 x SSC and 0.025% SDS, followed by 10 minutes at ambient temperature in 0.05 x SSC. The arrays were dried by brief centrifugation, placed back into the hybridization chamber and covered with new cover slips.

Each one of four microarrays was incubated with 30 µl of one of four different extension solutions (A, C, G or U) containing 1 x extension buffer (26 mM Tris-HCL pH 9.5, 6.5 mM MgCl_2_), 3.2 µM array-specific Cyanine-labeled dideoxynucleotides (ddNTP) and 6.6 U/µl thermosequenase DNA polymerase (USB Corporation). For example, extension solution A contained 400 nM Cyanine-3 ddATP, 400 nM Cyanine-5 ddATP, 800 nM Cyanine-3 ddCTP, 800 nM Cyanine-3 ddGTP and 800 nM Cyanine-3 ddUTP (Perkin Elmer, Waltham, MA). Extension solutions C, G and U had an analogous composition. Extension solutions were injected between the microarray slide and the cover slip and single base extension was allowed to proceed for ten minutes at 65°C. After a stringent wash for 10 minutes at 95°C in 1% SDS, microarrays were dried by centrifugation and scanned at 5 µm resolution on a GenePix 4000B scanner (Molecular Devices, Sunnyvale, CA) and analyzed using software developed in-house (see below).

### Data Analysis

Analysis of microarray data was performed using the open source software package MDAP, developed by the authors for microarray based mutation distribution analysis. (http://derisilab.ucsf.edu/software/MDAP/). P-values within the text were calculated using the Student's T-test, and both z-scores and t-tests were computed using the SciPy [33] software package.

## Supporting Information

Figure S1 Oligonucleotides of length 20 to 70 nucleotides were hybridized and extended with wild-type poliovirus template. Measurements were made for three genomic positions on four replicate arrays. The mean extension signal ± standard deviation (y-axis) is plotted against oligonucleotide length (x-axis).
(0.45 MB TIF)

Figure S2 (A) Oligonucleotides with a sequence at the 3′-end (red) that is complementary to a sequence elsewhere in the oligonucleotide (blue) can form dimers or hairpins as shown. Both structures can prime themselves in the absence of hybridized sample DNA, potentially leading to inappropriate extension (green). (B) A mutation at the position indicated in yellow disrupts extension for both the hairpin and dimmer. (C) Three examples of positions (genomic coordinates 2672, 2765, and 3284) in which oligonucleotide mutation prevented dimer-mediated extension. The background color represents the nucleotide expected to extend at the given position, and four foreground bars (some too short to be visible) represent the observed base-specific signals. For each position, unmodified oligonucleotide signal is shown on the left, and signal from the oligonucleotide modified to disrupt secondary structure is shown on the right.
(0.89 MB TIF)

Figure S3 (A) Extension from an oligonucleotide with an intact 3′ end, representing the signal observed for ‘correct’ incorporation. (B) Extension observed when the oligonucleotide is missing its 3′-most nucleotide. This generates a pattern where the mutation with the highest noise from a given oligonucleotide matches the expected signal from the 5′ neighboring oligonucleotide
(0.16 MB TIF)

Figure S4 Template from in vitro transcribed RNA was hybridized and extended on the surface of the array to determine oligonucleotide-specific noise levels. Any signal observed on oligonucleotides designed to assay mutations is assumed to derive from oligonucleotide-specific noise. Noise distributions are shown for oligonucleotides synthesized at Invitrogen's Hayward, CA facility in blue and the Frederick, MD facility in red. X-axis values are computed as described in equations 1–3 from the text, and y-axis values represent oligonucleotide counts.
(0.58 MB TIF)

Figure S5 Nucleotide-specific extension intensities are shown for (A) double stranded and (B) single stranded DNA samples prepared from in vitro transcribed poliovirus RNA. Y-axis values represent fluorescent signal intensities from Cy5 labeled ddNTPs, with a different labeled nucleotide added to each of four arrays as described in Figure 1. Only oligonucleotides templated with the expected wild-type nucleotide (denoted by color of data point) should be extended with Cy5 labeled ddNTPs in this homogeneous population. X-axis values represent fluorescence from extended Cy3 labeled ddNTPs, added as a mixture of all four nucleotides to each array. Since all oligonucleotides should extend Cy3 labeled ddNTPs, this signal is used as an indication of the total amount of extension per oligonucleotide. The signal-to-noise ratios are roughly equivalent to the distance between the two clusters of oligonucleotides observed in each graph.
(0.80 MB TIF)

Figure S6 Noise distribution graphs are shown for hybridization times of 12 hours, 5 hours, 4 hours, 2 hours, and 30 minutes. Line height represents the number of oligonucleotides showing the noise level specified on the x-axis. All data were generated from homogeneous in vitro transcribed poliovirus RNA samples.
(0.78 MB TIF)

Figure S7 To determine the effect of extension time on the signal-to-noise ratio of the array, single “A” arrays were hybridized with in vitro transcribed poliovirus RNA and extended for (A) 1 hour, (B) 20 minutes, and (C) 5 minutes. Each data point denotes the Cy5 (y axis), Cy3 (x axis), and expected extension base (color) of a single oligonucleotide. Signal-to-noise ratio of the full array is approximated here by the median distance between the “signal” cluster of oligonucleotides (red data points high on the y axis) and the “noise” cluster (green, blue, yellow data points).
(0.88 MB TIF)

Figure S8 The distribution of noise across all array oligonucleotides is shown. DNA from in vitro transcribed RNA was hybridized and extended on the array. Any signal corresponding to a base call different from the wild type poliovirus sequence was considered to be noise.
(0.46 MB TIF)

Figure S9 Normal single base extension from templated oligonucleotides (A) is affected by mismatches between the oligonucleotide and template (B). Mismatches near the 3′ end result in decreased signal and extension fidelity (C).
(0.08 MB TIF)

Figure S10 The difference between the average z-score for nonsynonymous mutations and synonymous mutations is shown on the y axis for each codon in the poliovirus capsid (x-axis). Values greater than zero indicate higher average significance of nonsynonymous mutations, and suggest positive selection. Values below zero indicate higher average significance of synonymous mutations, which suggests neutral mutation. No data was obtained for the indicated region of the capsid due to manufacturing defects in the oligonucleotides designed to assay that region.
(0.82 MB TIF)

Table S1 (0.03 MB DOC)

Table S2 (0.04 MB DOC)
