# Supplementary figures and images for: An Enhanced Single Base Extension Technique for the Analysis of Complex Viral Populations
Source: PLoS One. 2009 Oct 16;4(10):e7453. doi: 10.1371/journal.pone.0007453 (PMC2759544; doi:10.1371/journal.pone.0007453)

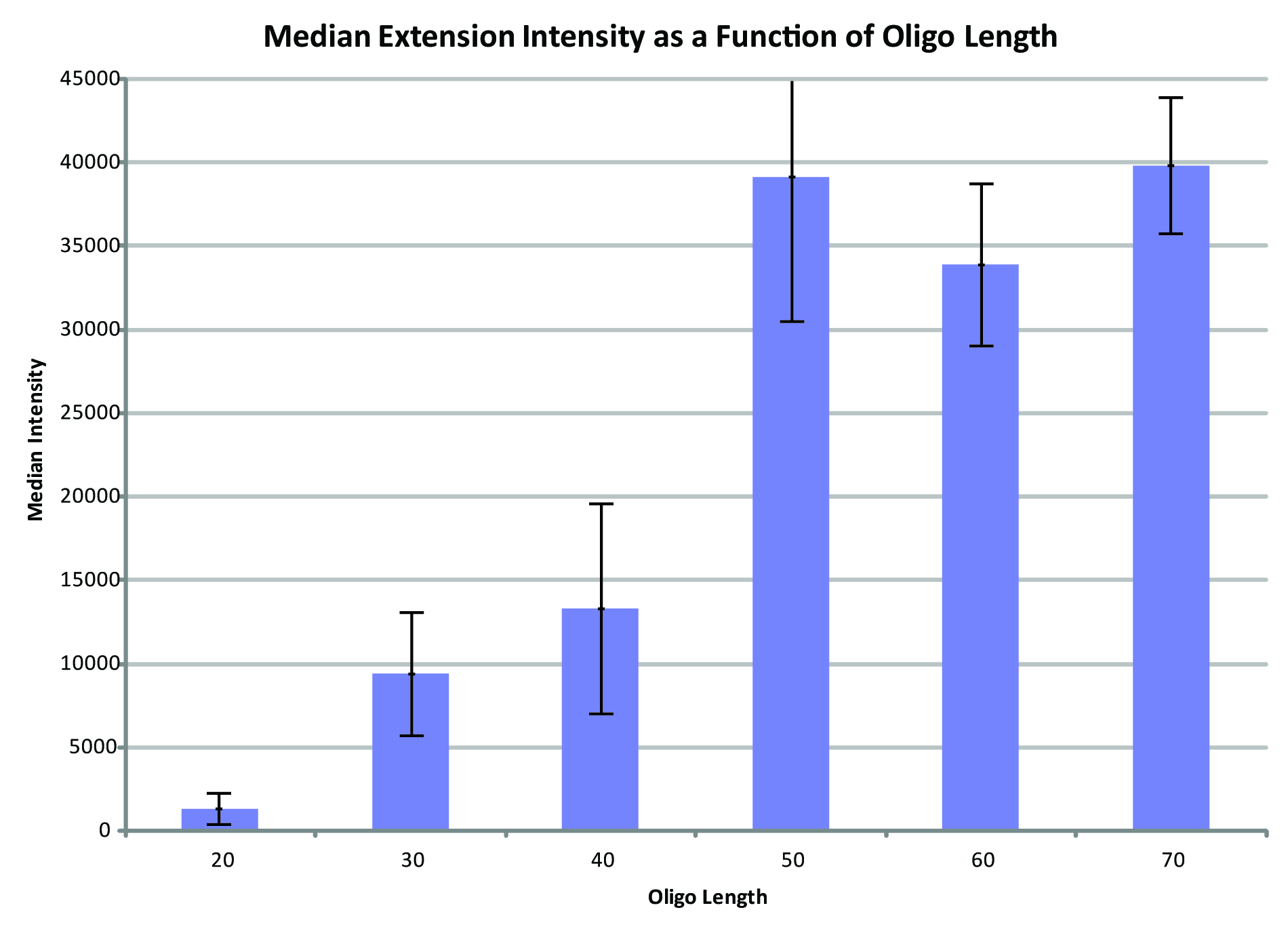

Supplement: Figure S1 — Oligonucleotides of length 20 to 70 nucleotides were hybridized and extended with wild-type poliovirus template. Measurements were made for three genomic positions on four replicate arrays. The mean extension signal ± standard deviation (y-axis) is plotted against oligonucleotide length (x-axis). (0.45 MB TIF) [file pone.0007453.s001.tif]

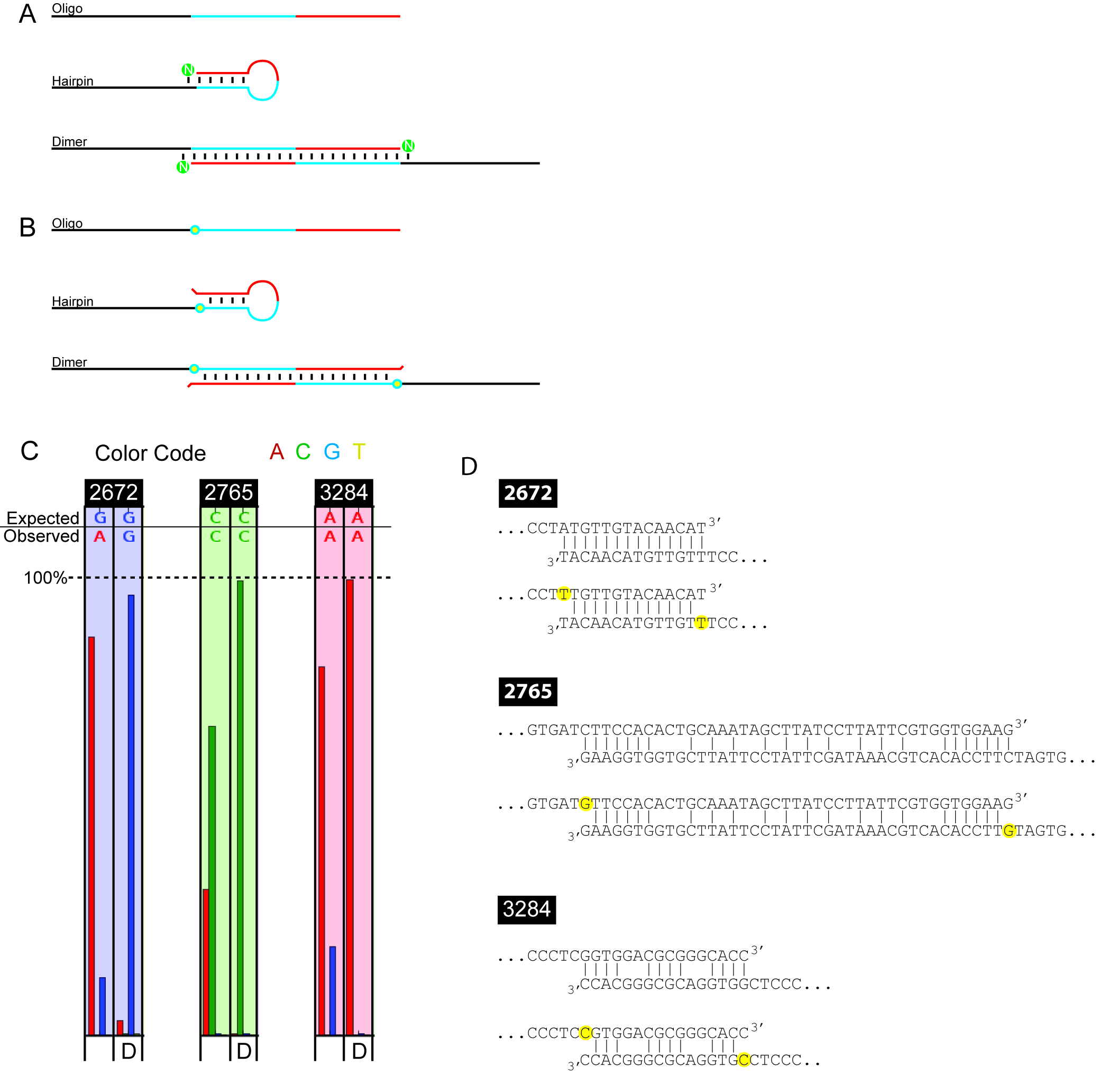

Supplement: Figure S2 — (A) Oligonucleotides with a sequence at the 3′-end (red) that is complementary to a sequence elsewhere in the oligonucleotide (blue) can form dimers or hairpins as shown. Both structures can prime themselves in the absence of hybridized sample DNA, potentially leading to inappropriate extension (green). (B) A mutation at the position indicated in yellow disrupts extension for both the hairpin and dimmer. (C) Three examples of positions (genomic coordinates 2672, 2765, and 3284) in which oligonucleotide mutation prevented dimer-mediated extension. The background color represents the nucleotide expected to extend at the given position, and four foreground bars (some too short to be visible) represent the observed base-specific signals. For each position, unmodified oligonucleotide signal is shown on the left, and signal from the oligonucleotide modified to disrupt secondary structure is shown on the right. (0.89 MB TIF) [file pone.0007453.s002.tif]

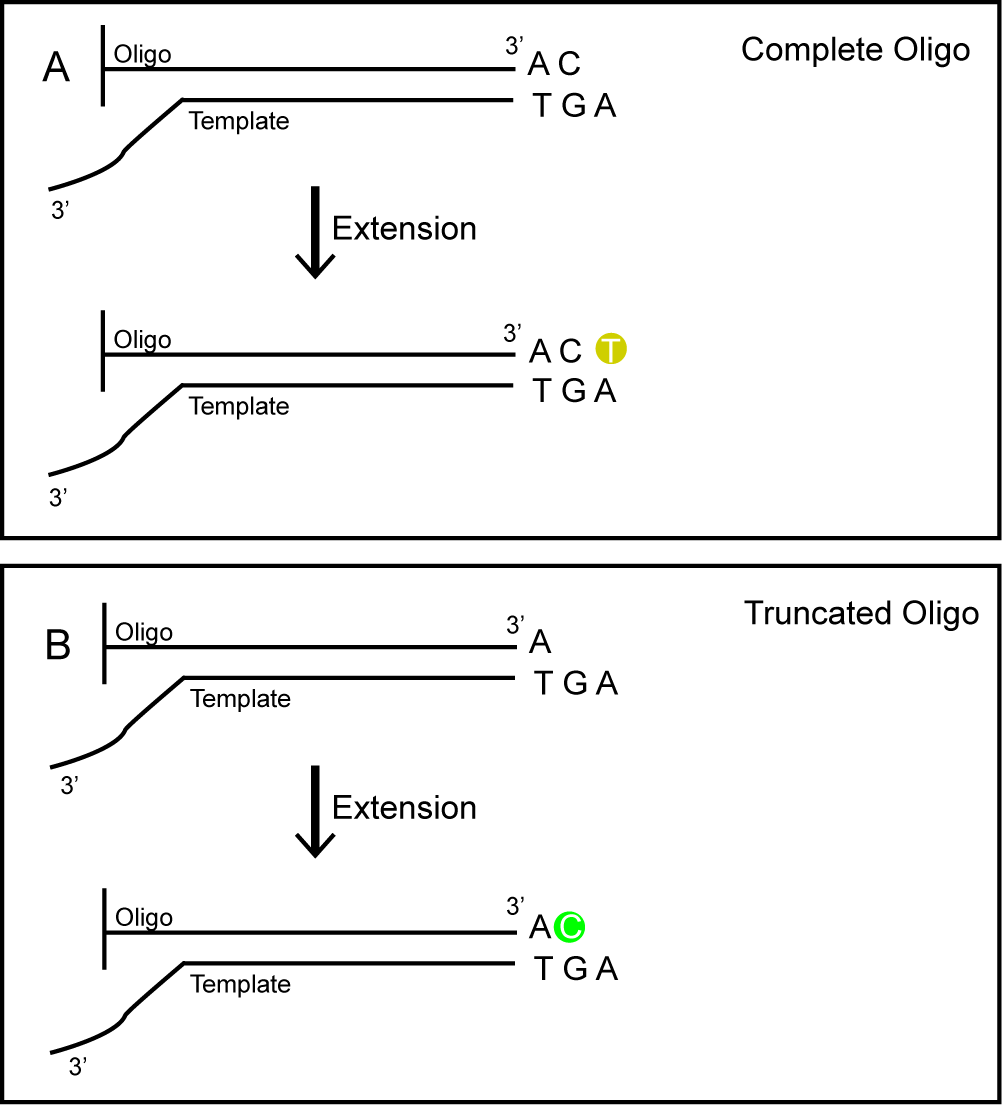

Supplement: Figure S3 — (A) Extension from an oligonucleotide with an intact 3′ end, representing the signal observed for ‘correct’ incorporation. (B) Extension observed when the oligonucleotide is missing its 3′-most nucleotide. This generates a pattern where the mutation with the highest noise from a given oligonucleotide matches the expected signal from the 5′ neighboring oligonucleotide (0.16 MB TIF) [file pone.0007453.s003.tif]

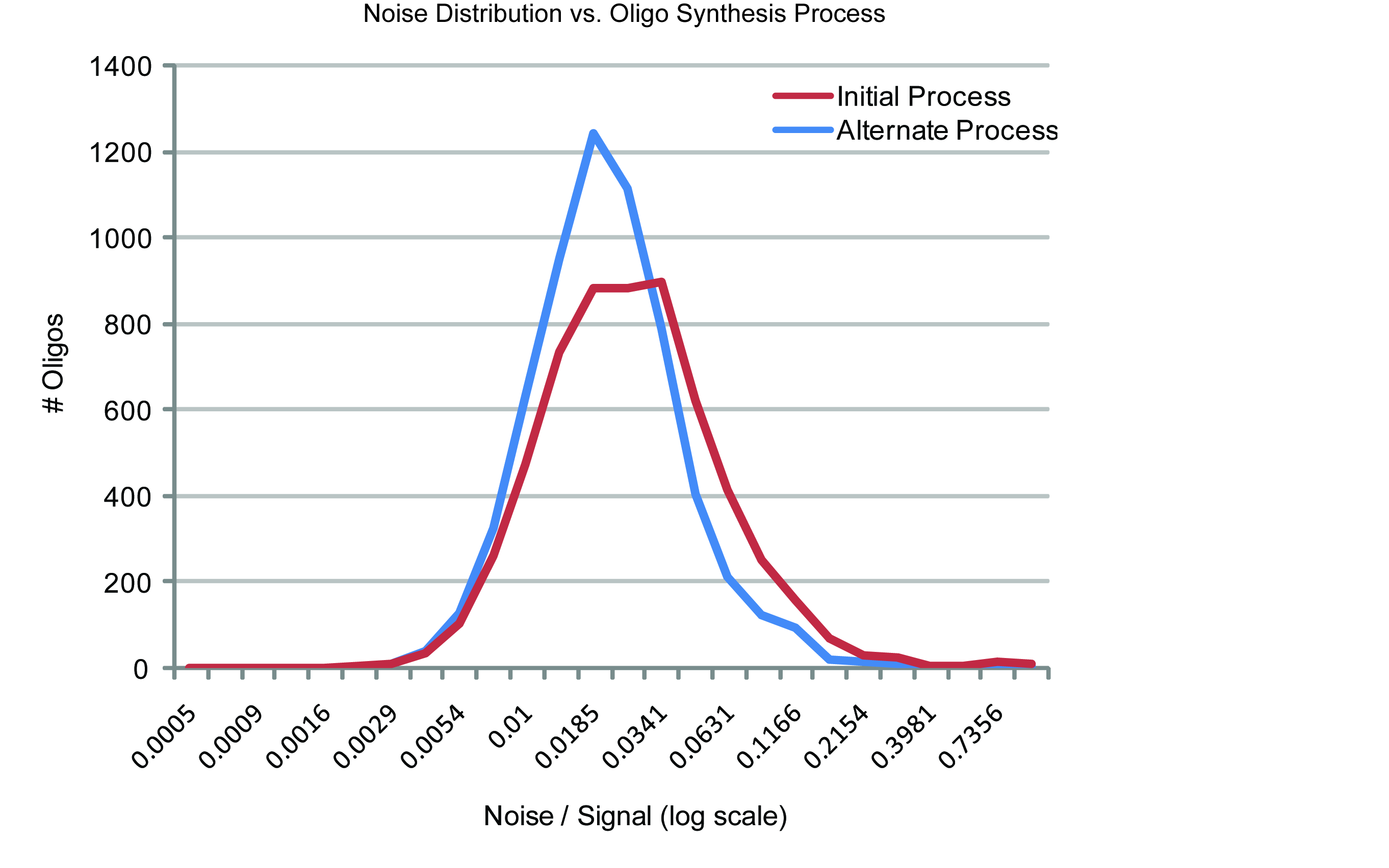

Supplement: Figure S4 — Template from in vitro transcribed RNA was hybridized and extended on the surface of the array to determine oligonucleotide-specific noise levels. Any signal observed on oligonucleotides designed to assay mutations is assumed to derive from oligonucleotide-specific noise. Noise distributions are shown for oligonucleotides synthesized at Invitrogen's Hayward, CA facility in blue and the Frederick, MD facility in red. X-axis values are computed as described in equations 1–3 from the text, and y-axis values represent oligonucleotide counts. (0.58 MB TIF) [file pone.0007453.s004.tif]

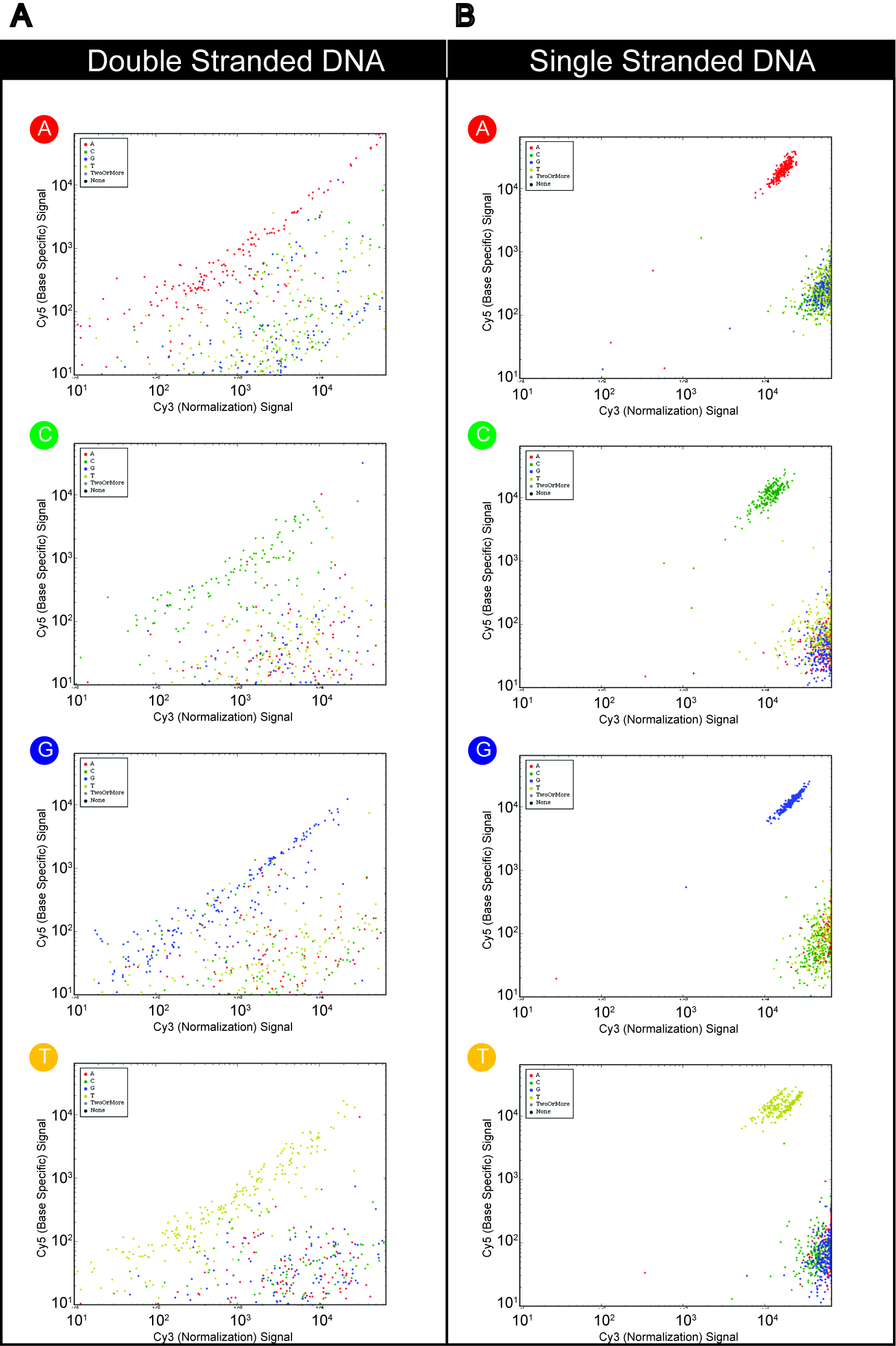

Supplement: Figure S5 — Nucleotide-specific extension intensities are shown for (A) double stranded and (B) single stranded DNA samples prepared from in vitro transcribed poliovirus RNA. Y-axis values represent fluorescent signal intensities from Cy5 labeled ddNTPs, with a different labeled nucleotide added to each of four arrays as described in Figure 1. Only oligonucleotides templated with the expected wild-type nucleotide (denoted by color of data point) should be extended with Cy5 labeled ddNTPs in this homogeneous population. X-axis values represent fluorescence from extended Cy3 labeled ddNTPs, added as a mixture of all four nucleotides to each array. Since all oligonucleotides should extend Cy3 labeled ddNTPs, this signal is used as an indication of the total amount of extension per oligonucleotide. The signal-to-noise ratios are roughly equivalent to the distance between the two clusters of oligonucleotides observed in each graph. (0.80 MB TIF) [file pone.0007453.s005.tif]

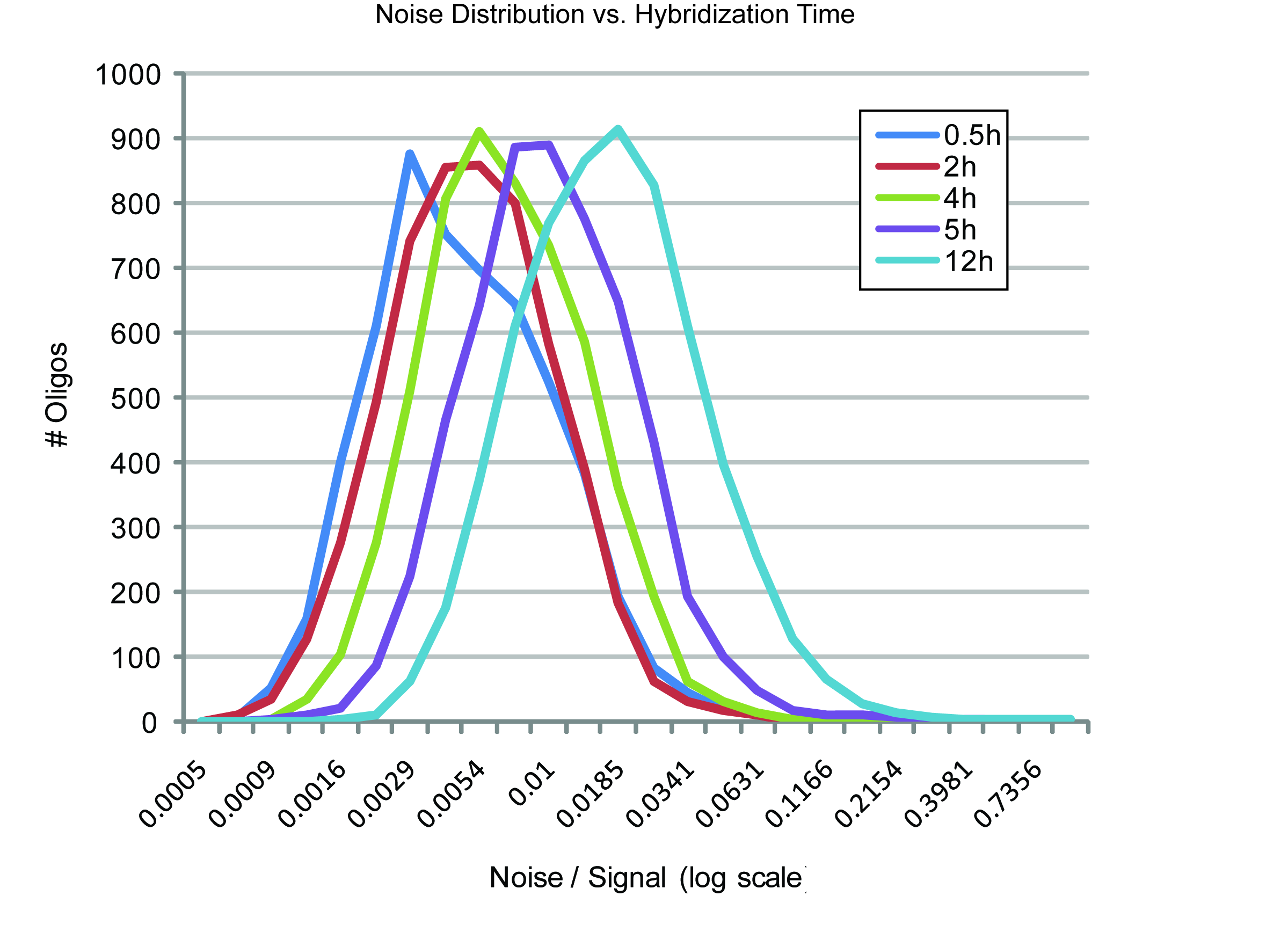

Supplement: Figure S6 — Noise distribution graphs are shown for hybridization times of 12 hours, 5 hours, 4 hours, 2 hours, and 30 minutes. Line height represents the number of oligonucleotides showing the noise level specified on the x-axis. All data were generated from homogeneous in vitro transcribed poliovirus RNA samples. (0.78 MB TIF) [file pone.0007453.s006.tif]

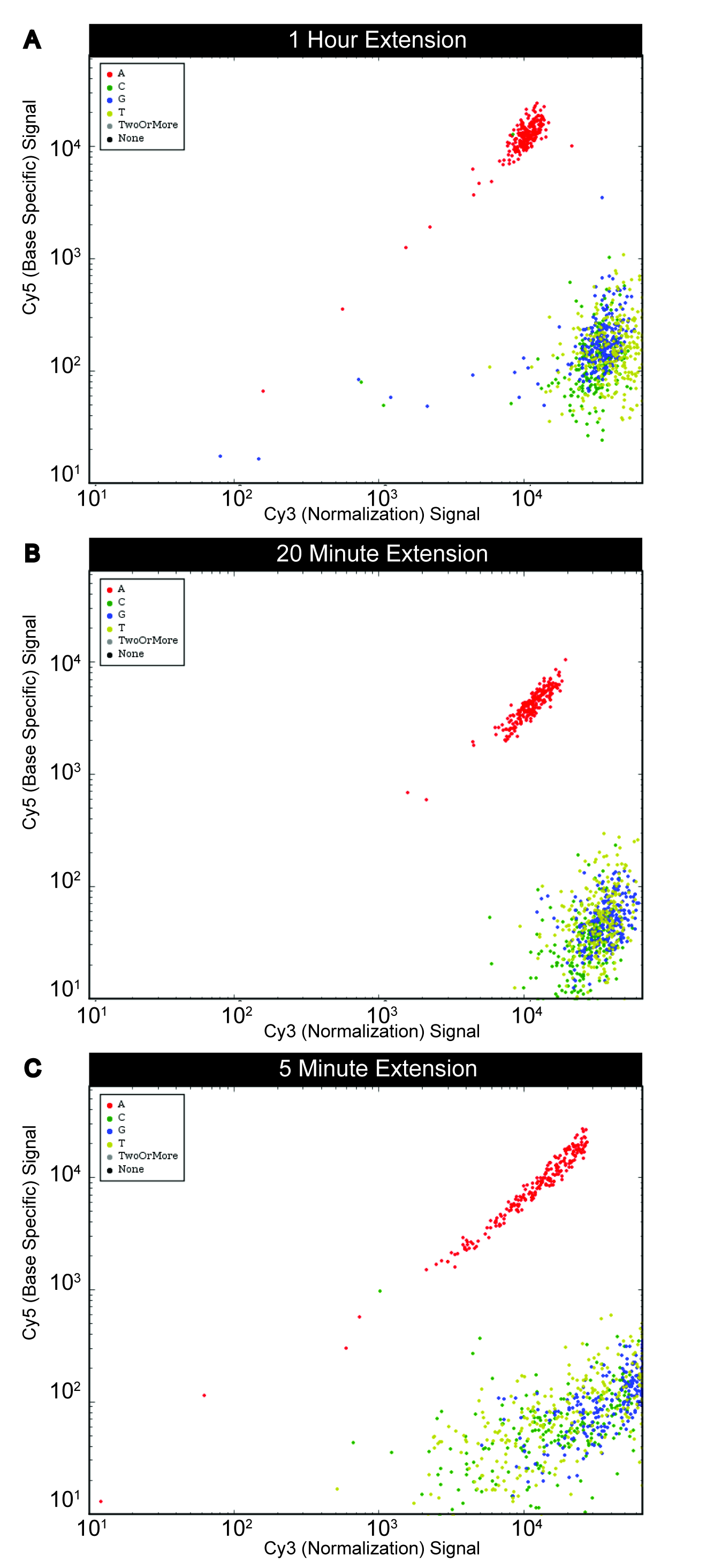

Supplement: Figure S7 — To determine the effect of extension time on the signal-to-noise ratio of the array, single “A” arrays were hybridized with in vitro transcribed poliovirus RNA and extended for (A) 1 hour, (B) 20 minutes, and (C) 5 minutes. Each data point denotes the Cy5 (y axis), Cy3 (x axis), and expected extension base (color) of a single oligonucleotide. Signal-to-noise ratio of the full array is approximated here by the median distance between the “signal” cluster of oligonucleotides (red data points high on the y axis) and the “noise” cluster (green, blue, yellow data points). (0.88 MB TIF) [file pone.0007453.s007.tif]

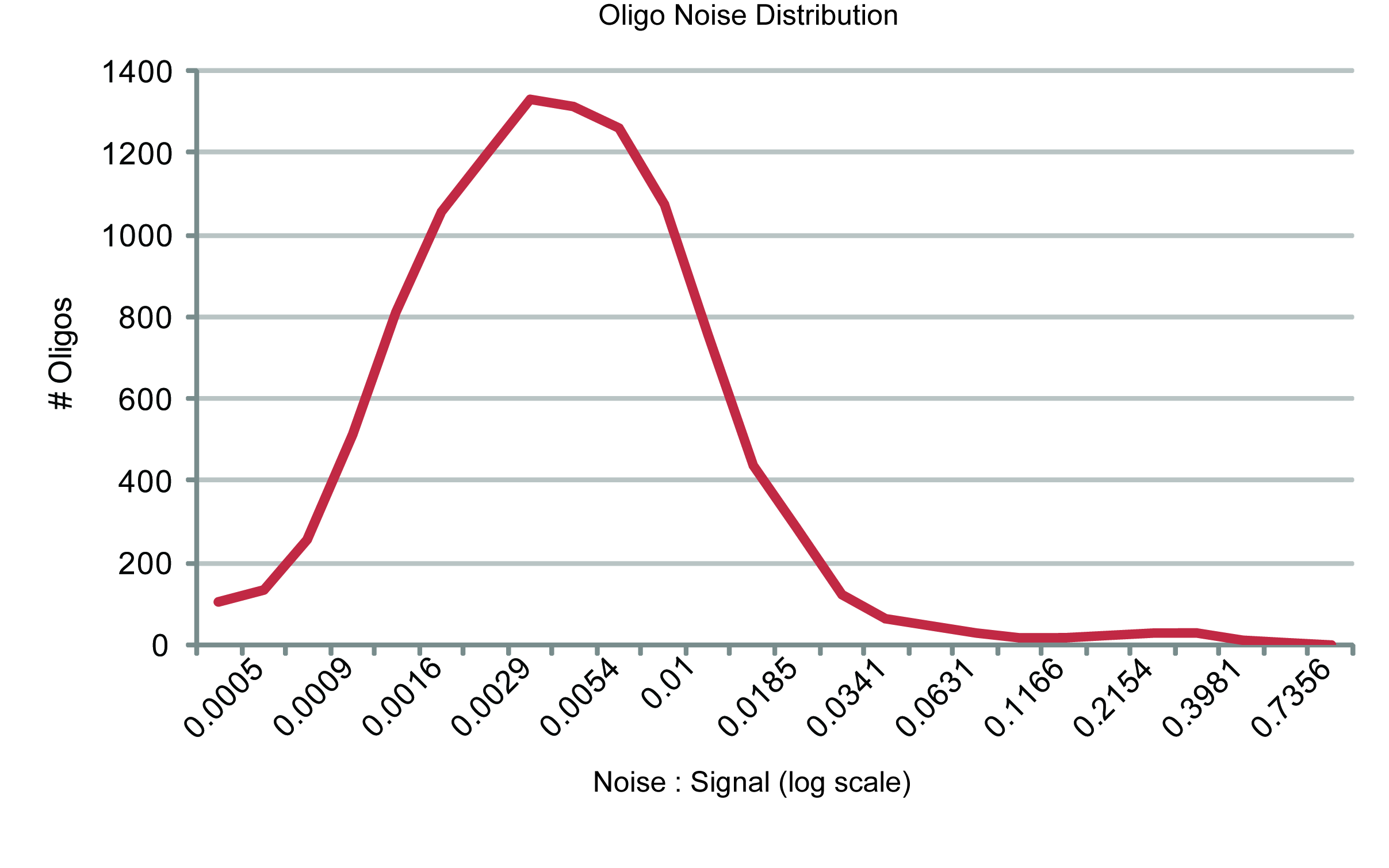

Supplement: Figure S8 — The distribution of noise across all array oligonucleotides is shown. DNA from in vitro transcribed RNA was hybridized and extended on the array. Any signal corresponding to a base call different from the wild type poliovirus sequence was considered to be noise. (0.46 MB TIF) [file pone.0007453.s008.tif]

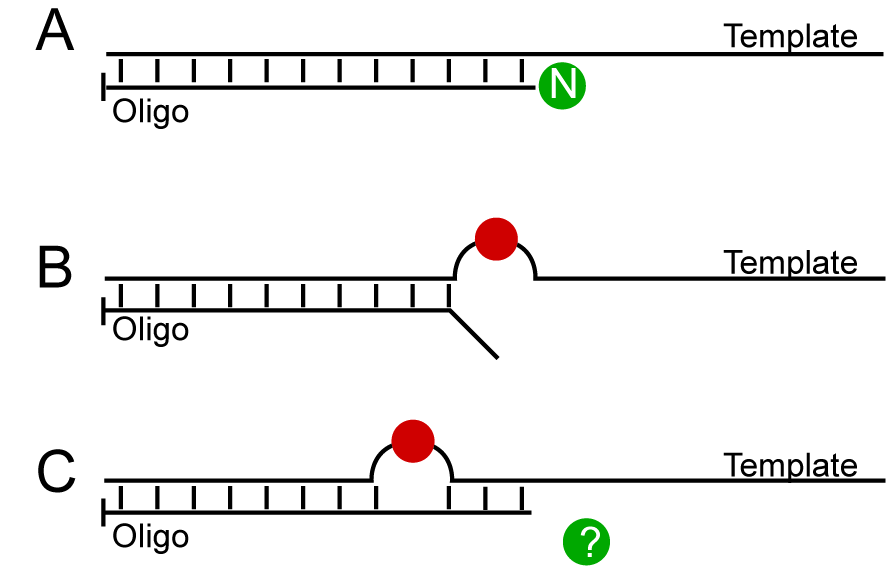

Supplement: Figure S9 — Normal single base extension from templated oligonucleotides (A) is affected by mismatches between the oligonucleotide and template (B). Mismatches near the 3′ end result in decreased signal and extension fidelity (C). (0.08 MB TIF) [file pone.0007453.s009.tif]

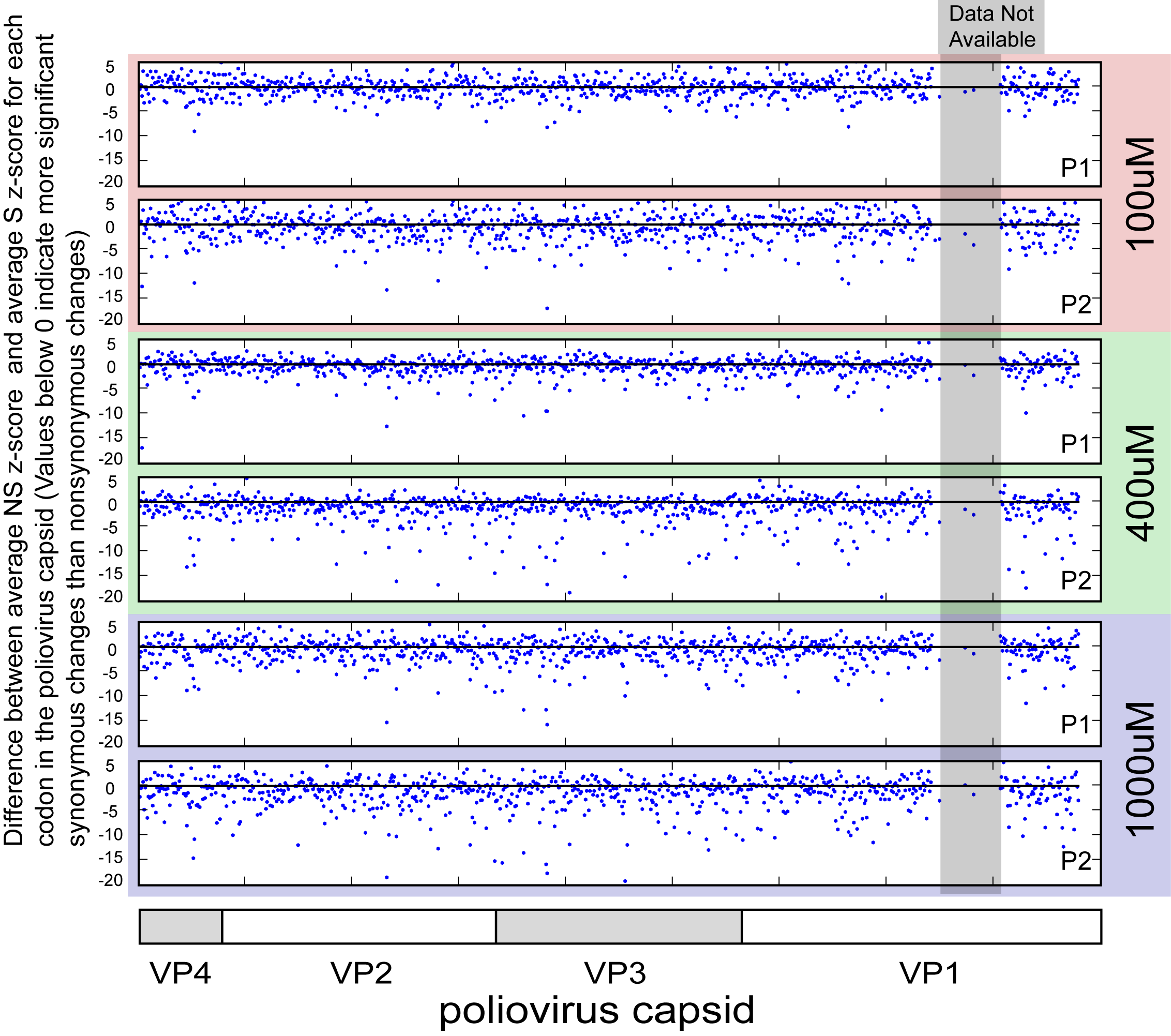

Supplement: Figure S10 — The difference between the average z-score for nonsynonymous mutations and synonymous mutations is shown on the y axis for each codon in the poliovirus capsid (x-axis). Values greater than zero indicate higher average significance of nonsynonymous mutations, and suggest positive selection. Values below zero indicate higher average significance of synonymous mutations, which suggests neutral mutation. No data was obtained for the indicated region of the capsid due to manufacturing defects in the oligonucleotides designed to assay that region. (0.82 MB TIF) [file pone.0007453.s010.tif]
