# Supplementary material for: An Enhanced Single Base Extension Technique for the Analysis of Complex Viral Populations
Source: PLoS One. 2009 Oct 16;4(10):e7453. doi: 10.1371/journal.pone.0007453 (PMC2759544; doi:10.1371/journal.pone.0007453)
Supplement: Table S1 — (0.03 MB DOC) [file pone.0007453.s011.doc]

**Table S1: Experimental Parameters Varied with Little or No Effect on Signal:Noise**

| HPLC Purification of Oligos |
| --- |
| Different Polymerases |
| Hybridization Temperature |
| Extension Temperature |
| Simultaneous Hyb + Extension |
| MAUI Hybridization |
| Template 3’ end blocking |
| Magnesium Concentration |
| Nucleotide Concentration |
| Nucleotide Ratios |
| Chemical Hot Start Extension |
| Physical Hot Start Extension |
| Cross Hybridization Prevention |
| RNA as Template |
| Hybridization in Glycerol |
