# Supplementary material for: An Enhanced Single Base Extension Technique for the Analysis of Complex Viral Populations
Source: PLoS One. 2009 Oct 16;4(10):e7453. doi: 10.1371/journal.pone.0007453 (PMC2759544; doi:10.1371/journal.pone.0007453)
Supplement: Table S2 — (0.04 MB DOC) [file pone.0007453.s012.doc]

**Table S2: Quasispecies Microarray Cost Breakdown**

| **Category** | **Component** | **Cost** |
| --- | --- | --- |
| **Array Design** | Oligos (7,441 x 70-mer) | $52,087 |
|  |  |  |
| **Array Fabrication** | Slides (200) | $2,400 |
|  | Microarrayer Use | $300 |
|  | **Total** | **$2,700** |
|  |  |  |
| **Per Sample Cost** | Array Fabrication (from above) | $27 |
|  | RNA Preparation | < $1 |
|  | cDNA Synthesis | $1 |
|  | PCR | $2 |
|  | IVT | $5 |
|  | Single-stranded template generation | $20 |
|  | Extension – Nucleotides | $20 |
|  | Extension – Polymerase | $20 |
|  | **Total** | **$95.00** |
